# Vitreous Decompression Combined with Phacoemulsification for Medically Unresponsive Acute Angle Closure

**DOI:** 10.1155/2021/5528281

**Published:** 2021-04-22

**Authors:** Xiaoli Xiang, Yuan Chen, Jinyu Wang, Zhengru Huang, Zheng Gu

**Affiliations:** Department of Ophthalmology, The Affiliated Changshu Hospital of Xuzhou Medical University, Changshu, China

## Abstract

The management of acute angle closure combined with an extremely shallow anterior chamber and cataract remains complex. This study evaluated a technique of vitreous needle aspiration combined with phacoemulsification for the treatment of acute angle closure with continuous high intraocular pressure (IOP). We retrospectively reviewed the results of vitreous needle aspiration combined with phacoemulsification in 17 eyes (17 patients) with acute angle closure with continuous high IOP and coexisting visually significant cataracts between September 2018 and April 2020 at the glaucoma unit of the affiliated Changshu Hospital of Xuzhou Medical University. The main outcomes were the best corrected visual acuity (BCVA), IOP, anterior chamber depth (ACD), angle open distance 500 (AOD500), number of antiglaucoma medications, and surgery-associated complications. There were no complications during phacoemulsification and a foldable acrylic intraocular lens was implanted in the capsular bag in all 17 patients. For all patients, vitreous needle aspiration was successful at the first attempt. The BCVA improved from 2.02 ± 0.54 logMAR preoperatively to 0.73 ± 0.57 logMAR postoperatively at the final examination (*p* < 0.001). The mean IOP was 54.47 ± 5.33 mmHg preoperatively and 15.59 ± 2.35 mmHg at the final examination (*p* < 0.001) without any medication. The ACD was 1.70 ± 0.16 mm preoperatively and 3.35 ± 1.51 mm at the final examination (*p* < 0.001). The AOD500 was 0.07 ± 0.02 mm preoperatively and 0.51 ± 0.04 mm at the final examination (*p* < 0.001). Our vitreous needle aspiration technique can be performed safely in phacoemulsification for the management of acute angle closure with continuous high IOP.

## 1. Introduction

Angle closure disease involves closure of the ocular drainage angle, thereby blocking aqueous humor from being discharged through the trabecular meshwork [1]. It results in the elevation of the intraocular pressure (IOP), followed by the development of glaucomatous optic neuropathy [2]. Risk factors for angle closure disease are a shorter axial length, shallow anterior chamber, thick peripheral iris roll, and thick lens in the anterior position [3]. Its subtypes include primary angle closure, suspect, acute angle closure (AAC), and primary angle closure glaucoma [4].

AAC is an ophthalmic emergency characterized by the sudden closure of the anterior chamber angle followed by a rapid rise in the IOP to high levels [5]. Patients with AAC require emergency management to avoid blindness. The EAGLE study supported the notion that, in general, early or clear lens extraction is an acceptable therapeutic approach to prevent AAC glaucoma [6]. Additionally, phacoemulsification with intraocular lens implantation results in a reduction in the IOP, as well as a significant reduction in the number of glaucoma medications. Thus, phacoemulsification is increasingly used for the primary management of AAC [7, 8]. Although surgery is often challenging (e.g., due to a shallow anterior chamber, synechiae, or weak zonules [7]), the results are encouraging, with significant improvement in visual acuity; however, this improvement is less in patients with primary angle closure glaucoma than in patients with primary angle closure [7].

The principle of treatment is to reduce the IOP before surgery. However, in some patients with AAC, the IOP cannot be effectively reduced by nonsurgical conservative treatment. Therefore, we retrospectively reviewed the results of vitreous needle aspiration combined with phacoemulsification in cases of AAC and assessed its safety and efficacy as an aid to the surgical management of this common emergent condition.

## 2. Methods

This study was approved by the Institutional Review Board of the affiliated Changshu Hospital of Xuzhou Medical University (Changshu, China). The study was conducted in accordance with the Helsinki Declaration. Informed consent for the operation was obtained from all patients.

### 2.1. Patients

A retrospective case series was conducted. Consecutive patients with AAC treated between September 2018 and April 2020 at the glaucoma unit of the affiliated Changshu Hospital of Xuzhou Medical University (Changshu, China) underwent vitreous needle aspiration combined with phacoemulsification. The inclusion criteria were as follows: acute angle closure complicated with cataract, IOP uncontrolled by 2-3 days of drug treatment, and a very shallow anterior chamber. Preoperative treatment included pilocarpine, topical antiglaucoma drops, oral carbonic anhydrase inhibitors, and mannitol. Exclusion criteria were as follows: uncontrolled ocular infection, significant opacity of the cornea, severe systemic disease (such as cardiac insufficiency, renal insufficiency, and cerebrovascular accident), and inability to complete scheduled follow-ups.

### 2.2. Assessments

All eligible patients underwent a thorough eye examination before the intervention, including best corrected visual acuity (BCVA), noncontact tonometry, and slip-lamp examination. The BCVA was measured by a decimal chart and was converted to logMAR for computing purposes [9]. Axial length was examined by A-scan ultrasonography. The vitreous and retina were examined by B-scan ultrasonography. The anterior chamber angle measured as the angle-opening distance at 500 *μ*m (AOD500) and anterior chamber depth (ACD) were examined by ultrasound biomicroscopy (QUANTEL AVISO YM0020495, Cournon d'Auvergne, France). The ACD was defined as the distance between the posterior corneal surface and the anterior lens surface [10]. The power of the postchamber intraocular lens was measured. The history of antiglaucoma medication was recorded.

All patients were followed up at least 6 months postoperatively. Ocular parameters, including IOP, BCVA, complications, and medications, were recorded at each visit. The ACD and AOD500 were also measured by ultrasound biomicroscopy at least two weeks postoperatively.

### 2.3. Surgical Procedures

All surgeries were performed under peribulbar anesthesia by one experienced ophthalmologist (Z.H). Compound tropicamide eye drops were used to dilate the pupil every 10 minutes, starting 30 minutes before surgery. After topical anesthesia, 1% lidocaine was injected into the subconjunctival.

**Figure 1:**
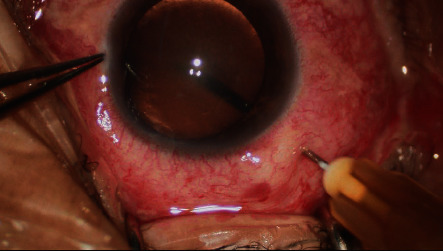
Vitreous aspiration was performed using a 27 gauge needle attached to a 1 ml syringe.

Vitreous needle aspiration was performed before phacoemulsification in all patients. Vitreous aspiration was performed using a 27 gauge needle attached to a 1 ml syringe, 3.5 mm from the limbus, vertically inserted through the pars plana (Figure 1). The vitreous extraction was no more than 0.2 ml. If the eye became too firm or the chamber was still too shallow, the limited vitreous aspiration was repeated.

After vitreous needle aspiration, a temporal corneal incision of 2.8 mm in width and one side port were created. The anterior chamber was filled with a cohesive ophthalmic viscoelastic device and a continuous curvilinear capsulorhexis of 5-6 mm in diameter was completed. Phacoemulsification was performed using the phaco chop technique with an Infiniti phacoemulsification machine. Irrigation aspiration of the remaining cortical material and foldable intraocular lens implantation in the capsular bag was achieved in all cases.

Postoperatively, tobramycin, dexamethasone eye drops (TobraDex, S.A., Alcon Couvreur N.V., Belgium), and nonsteroidal anti-inflammatory eye drops (Pranolulin, Senju Pharmaceutical Co., Ltd., Fukusaki Plant, Japan) were administered four times daily for 4–6 weeks consecutively. Antiglaucoma eye drops or oral medicine was administered if necessary.

### 2.4. Statistical Analysis

Data are reported as mean ± standard deviation or as a number (percentage). Differences between preoperative and postoperative values were evaluated using the paired t-test. Statistical analyses were performed using the SPSS 19.0 statistical software (SPSS Inc., Chicago, IL, USA), and *p* values <0.05 were considered significant.

## 3. Results

### 3.1. Baseline Characteristics

In total, 17 eyes from 17 patients were included in this study. The average age was 69.8 ± 6.4 years (range, 55–85 years) and 13 patients (76.5%) were female. The average time of eye discomfort was 4.59 ± 2.74 days (range, 1–10 days). The right eye was involved in 10 cases (58.8%), and the left eye was involved in 7 cases (41.2%).

### 3.2. Outcomes

**Figure 2:**
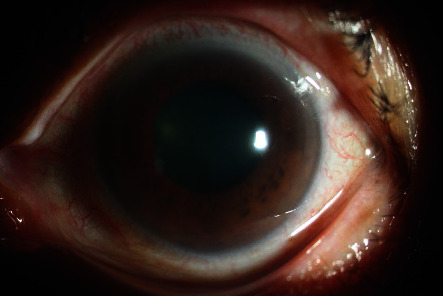
Preoperative image of a patient with acute angle closure. The preoperative treatment included pilocarpine, topical antiglaucoma drops, oral carbonic anhydrase inhibitors, and mannitol.

**Figure 3:**
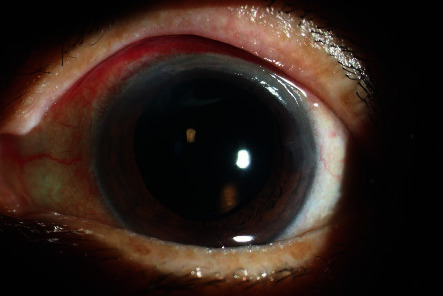
Postoperative image of the same patient as that in Figure 2.

**Figure 4:**
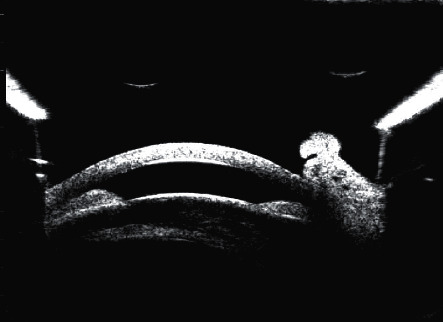
Preoperative ultrasound biomicroscopic image of a patient with acute angle closure.

**Figure 5:**
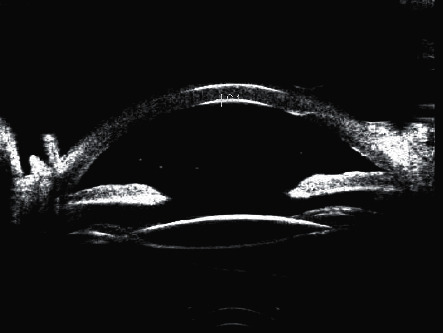
Postoperative ultrasound biomicroscopic image of the same patient as that in Figure 4.

For all patients, vitreous needle aspiration was successful at the first attempt. There were no complications during phacoemulsification, and a foldable acrylic intraocular lens was implanted in the capsular bag in all 17 patients. One patient had mild striate keratopathy on the first postoperative day, which resolved completely within 3 days. None of the patients required antiglaucoma medications in the follow-up period. Representative preoperative and postoperative images are shown in Figures 2–5.

In all patients, the postoperative IOP was in the normal range. The mean IOP significantly decreased from 54.47 ± 5.33 mmHg (range, 43–60 mmHg) preoperatively to 15.59 ± 2.35 mmHg (range, 11–19 mmHg) at the final examination, without any medication (*p* < 0.001). The BCVA significantly improved from 2.02 ± 0.54 logMAR preoperatively to 0.73 ± 0.57 logMAR at the final examination (*p* < 0.001). The mean ACD significantly increased from 1.70 ± 0.16 mm (range, 1.17–1.94 mm) to 3.35 ± 1.51 mm (range, 3.08–3.66 mm) at the final examination (*p* < 0.001). The mean AOD500 significantly increased from 0.07 ± 0.02 mm (range, 0.03–0.11 mm) to 0.51 ± 0.04 mm (range, 0.42–0.56 mm) at the final examination (*p* < 0.001).

## 4. Discussion

Acute primary angle closure is an important cause of blindness in East Asia [11]. In China, an estimated 28 million individuals have occludable drainage angles [12]. The risk of developing AAC is three times higher in women than in men [7]. Consistent with this, women comprised 76.5% of our study population. In addition to the effect of race, the incidence of AAC rises steeply with age, which may result from age-related increases in lens thickness, decreased ACD, and an anteriorly moved lens center [13]. A significant proportion of patients with AAC do not respond adequately to medical treatment alone. In such cases, it is important to understand how to perform the surgery safely and effectively [14].

Intraoperative procedures, such as corneoscleral wound incision, capsulorhexis, cortex aspiration, and insertion of the foldable intraocular lens, are difficult to perform in eyes with a shallow anterior chamber. A shallow chamber poses an increased risk of endothelial cell loss, as the phaco tip is closer to the endothelium during nucleus emulsification [15]. Endothelial cell loss, iris damage, intraoperative pupillary constriction, capsulorhexis tear or capsular rupture, subluxation of lens material, and vitreous loss may be occur red during the phacoemulsification in AAC because of a shallow anterior chamber and positive pressure [15]. Although limited pars plana vitrectomy is currently considered to be the only way to successfully deepen the anterior chamber [16], vitreous tap using needle aspiration is a simple and effective alternative.

The main risk involved in our technique is the potential for vitreous traction [15]. Some scholars believe that the use of a vitrectomy cutter to remove vitreous decreases the potential for vitreous traction compared to a needle aspiration technique [15]. However, in a large, multicenter study on endophthalmitis vitrectomy, there was no significant difference between vitreous needle aspiration and automated vitrectomy regarding posterior segment complications and the final visual outcome over a follow-up period of 9–12 months [17]. Additionally, our patients were older and had posterior vitreous detachment and liquefaction; therefore, the vitreous fluid could be successfully extracted without vitreous traction and other complications. Furthermore, a rapid breakthrough of the vitreous cortex into the liquefied vitreous lacuna during puncture is key to success. In the present study, vitreous puncture with vitreous needle aspiration was performed to remove 0.2 ml of vitreous; because of the small volume of the aspiration, the vitreous could be successfully aspirated in most cases without increasing the risk of retinal traction. Although it could lead to posterior displacement of the lens, this technique deepened the anterior chamber, decreased the IOP and positive vitreous pressure, and reduced the chance of posterior capsule rupture and suprachoroidal hemorrhage.

Considering the cost of the surgery and the potential cardiovascular and respiratory risks, we did not select general anesthesia. Instead, we injected lidocaine under the conjunctiva after topical anesthesia because it did not increase the vitreous and orbital pressures. Furthermore, common needles and syringes were used in the aspiration; there was no need to use expensive vitrectomy supplies. In the developing world, where healthcare institutions impose strict controls on cataract surgery costs, the use of vitrectomy increases the cost. In contrast, our technique reduced the operative time and surgical scope, did not require suturing of the puncture site, and created favorable surgical conditions for phacoemulsification. Increased anterior chamber space and decreased IOP render uneventful cataract phacoemulsification possible and reduce the risk of corneal endothelium injury. Consistent with the previous literature [18], the vitreous was successfully extracted on the first attempt, the phacoemulsification surgery was successfully completed, the postoperative vision was improved, and antiglaucoma drugs were not required to maintain the IOP within the normal range for all patients in the current study.

## 5. Conclusions

Our vitreous needle aspiration technique can be performed safely in phacoemulsification for the management of AAC with continuous high IOP. This technique can significantly reduce the risk of intraoperative complications and operative costs; however, further long-term studies with a larger sample size are required.

## Data Availability

The datasets generated and/or analyzed during the current study are not publicly available due to privacy concerns but are available from the corresponding author on reasonable request.
